# De novo tissue formation using custom microporous annealed particle hydrogel provides long-term vocal fold augmentation

**DOI:** 10.1038/s41536-023-00281-8

**Published:** 2023-02-23

**Authors:** Lauren J. Pruett, Hannah L. Kenny, William M. Swift, Katarina J. Catallo, Zoe R. Apsel, Lisa S. Salopek, Philip O. Scumpia, Patrick S. Cottler, Donald R. Griffin, James J. Daniero

**Affiliations:** 1 Department of Biomedical Engineering, University of Virginia, Charlottesville, VA 22903 USA; 2 School of Medicine, University of Virginia, Charlottesville, VA 22903 USA; 3 Department of Otolaryngology-Head and Neck Surgery, University of California, Davis, CA 95616 USA; 4 Department of Plastic Surgery, University of Virginia, Charlottesville, VA 22903 USA; 5 Department of Medicine, Division of Dermatology and Department of Pathology, Division of Dermatopathology, University of California, Los Angeles, CA 90095 USA; 6 Department of Chemical Engineering, University of Virginia, Charlottesville, VA 22903 USA; 7 Department of Otolaryngology-Head and Neck Surgery, University of Virginia, Charlottesville, VA 22903 USA

**Keywords:** Tissues, Implants

## Abstract

Biomaterial-enabled *de novo* formation of non-fibrotic tissue in situ would provide an important tool to physicians. One example application, glottic insufficiency, is a debilitating laryngeal disorder wherein vocal folds do not fully close, resulting in difficulty speaking and swallowing. Preferred management of glottic insufficiency includes bulking of vocal folds via injectable fillers, however, the current options have associated drawbacks including inflammation, accelerated resorption, and foreign body response. We developed a novel iteration of microporous annealed particle (MAP) scaffold designed to provide persistent augmentation. Following a 14-month study of vocal fold augmentation using a rabbit vocal paralysis model, most MAP scaffolds were replaced with tissue *de novo* that matched the mixture of fibrotic and non-fibrotic collagens of the contralateral vocal tissue. Further, persistent tissue augmentation in MAP-treated rabbits was observed via MRI and via superior vocal function at 14 months relative to the clinical standard.

## Introduction

**Fig. 1:**
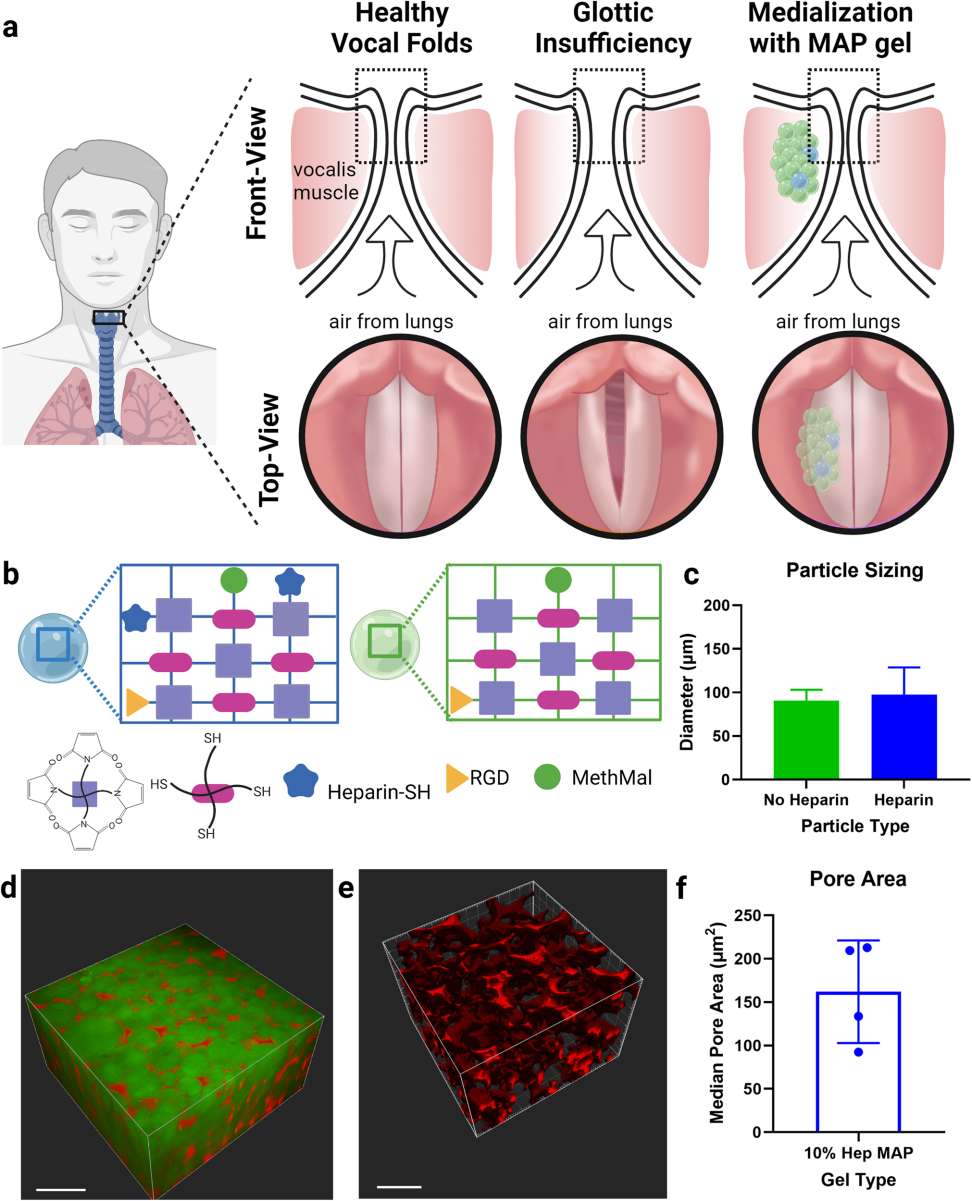
MAP application for glottic incompetence. **a** Schematic of healthy vocal folds, glottic insufficiency and MAP gel augmentation of the left vocal fold. **b** Schematic of heparin and no heparin particles which are used in the vocal MAP gel formulation. **c** Particle sizes for the heparin and no heparin gel populations which are mixed in the final heterogeneous formulation (10% heparin microgels). **d** Fluorescent dextran (red) infused into the MAP gel (green) to visualize porosity. **e** Pore rendering generated by Imaris software. **f** Pore cross sectional area analysis. Individual data points represent median pore areas. Data is represented as mean+/− standard deviation.

Glottic insufficiency is a debilitating laryngeal disorder in which the loss of tissue volume prevents vocal folds from fully closing, producing a glottic gap (Fig. 1a) that results in difficulty speaking and swallowing^1^. Patients that do not respond to conservative management with voice therapy must undergo augmentation of the vocal folds with a tissue filler to restore volume. Injectable biomaterials are desirable for vocal fold augmentation over implantation surgeries due to their minimally invasive delivery^2^. However, injectable fillers for vocal fold augmentation have been adapted from temporary dermal fillers used in cosmetic plastic surgery that only offer a transient benefit^3^.

A variety of biomaterial approaches have been explored both preclinically and clinically to address the needs of glottic insufficiency, focused on avoiding significant inflammation and increasing longevity of the implant^1,3–5^. The natural biomaterials explored—including hyaluronic acid, gelatin, and collagen—have shown a limited inflammatory response; however, they have also displayed undesirable properties for vocal fold augmentation, including quick resorption within 4–6 months, migration within the tissue, and requirement of follow-up surgeries in a patient’s lifetime^1,3,6–9^. Scaffolds developed for long-term tissue augmentation often consist of inflammatory microparticles embedded in a degradable gel carrier^10–12^, including a clinically approved material consisting of calcium hydroxyapatite particles in a carboxymethylcellulose carrier^2^. Unfortunately, these scaffolds rely on sustained tissue inflammation in the form of a foreign body response (FBR)^10^ around the individual microparticles to create intentional fibrosis (i.e., tissue scarring) to provide tissue bulking for longer durations. Even with these fibrotic approaches, once the FBR subsides patients often still require follow-up surgeries after 2 years^3^. The ideal injectable would be able to provide augmentation permanently by induction of a non-FBR dependent local tissue formation to maintain volume^13^, while retaining the ability to be injected with standard clinical apparatus^5^.

We have developed a unique formulation of microporous annealed particle (MAP) hydrogel based on minimally degradable materials, without enzymatically or hydrolytically susceptible bonds^14^ (e.g., esters, thioesters), to address this clinical need^15^. As a biomaterial platform, MAP gel is defined as an injectable slurry of individual spherical microgels which undergo a secondary covalent inter-particle crosslinking step to form a structurally stable scaffold with interconnected porosity resulting from imperfect packing of spherical microgels^16^. In our initial published study of MAP hydrogel for vocal fold augmentation in an uninjured rabbit model, we saw material permanence out to 6 months with significant cell infiltration and no histologically discernible foreign body response^15^. This motivated us to perform an extended study in a clinically relevant model of glottic insufficiency^17^ described in this manuscript.

## Results

### Synthesis and characterization of particle building blocks for a minimally degradable porous scaffold

For this study, we used a fully PEG-based MAP formulation consisting of a PEG-maleimide backbone and PEG-thiol crosslinker like our previous study^15^. This formulation was modified slightly with the addition of a custom annealing macromer^18^ to decrease the time of light exposure needed for scaffold annealing, and the incorporation of heparin microislands (i.e., microgels including immobilized heparin) to accelerate tissue integration into the scaffold via the ability to sequester endogenous growth factors^19^ (Fig. 1b). To characterize our vocal MAP gel formulation, we performed mechanical and pore size analysis. Due to the critical importance of biomechanical properties of the vocal fold for vocal function, we matched our gel formulations (heparin and no heparin) to 14 kPa via Instron compressive testing to match vocal fold stiffness (Supplementary Fig. 1), which is not currently matched by the current clinical treatment^15^. Further, for the heterogeneous MAP composition used (10% heparin microislands), both microgel populations (heparin and no heparin) were matched in mechanical stiffness and particle size to each other. Importantly, MAP gel’s interconnected porosity (Fig. 1d) allows for significant cell infiltration that does not rely on material degradation like traditional sol-gel hydrogels. By infusing our vocal MAP scaffold pores with fluorescently labeled dextran in vitro (Fig. 1e), we quantified median pore cross-sectional areas of 162 µm^2^ (Fig. 1f), which corresponds to a pore diameter of 14.4 µm in our scaffold composed of microgels approximately 95 µm in diameter (Fig. 1e).

### Execution of a rabbit model of glottic insufficiency

In this extended study, we employed a vocal fold paralysis model to assess the potential of our permanent MAP formulation to maintain volume and restore function for 14 months. Briefly, to establish the model of glottic insufficiency, New Zealand white rabbits had >2 cm segment of the left recurrent laryngeal nerve resected (note: vocal fold denervation is a common clinical cause of glottic insufficiency^20^) (Supplementary Fig. 3). This allowed for the left hemilaryngeal musculature, including the vocalis portion of the thyroarytenoid muscle, to atrophy for 3 months prior to treatment resulting in complete paralysis of the left vocal fold. After paralysis, we treated the glottal insufficiency via injection augmentation of the paralyzed vocal fold (left) in rabbits with MAP gel or hyaluronic acid (Restylane-L) as a clinical control (*n* = 6). Hyaluronic acid was chosen as the clinical control due to it being the most used clinical material in an outpatient setting, which would parallel the use of MAP gel. This head-to-head study design compares the vocal formulation of MAP gel with the current clinical standard treatment for vocal fold augmentation. We were specifically interested in determining the timeline of augmentation retention, vocal function outcomes, and the long-term tissue response to the implant.

### Measurement of Airway Volume Maintenance via MRI

**Fig. 2:**
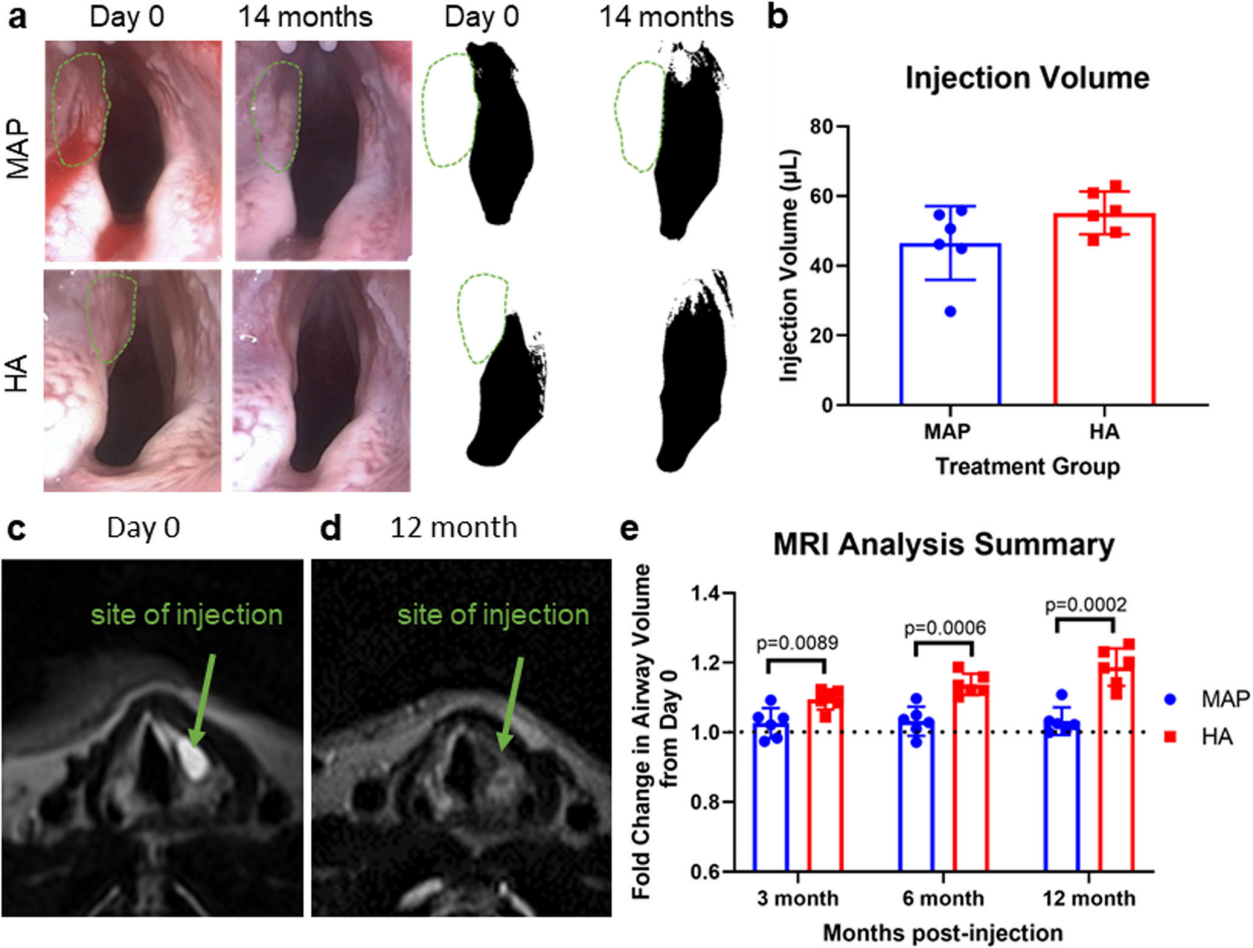
Volume retention. **a** Endoscopic visualization of augmentation at day 0 and 14 months for both treatment groups, with a green outline indicating the site of augmentation. Contrast images for each group show the outline of the airway along with the injection placement. **b** MRI calculated injection volumes of each treatment group. **c** MRI visualization of MAP implant at Day 0. **d** 12-month MRI showing durable augmentation. **e** MRI analysis of airway volume across 12 months between MAP and HA groups. Data is represented as mean+/− standard deviation. Statistics: Unpaired student’s *t*-test. *n* = 6.

To quantify scaffold volume maintenance, we performed MRI on the rabbits immediately after injection and at 3, 6, and 12 months to evaluate airway volume throughout the study. At day 0, we were able to distinguish both biomaterials due to the high-water content of each material, producing increased T2 signal intensity, (Fig. 2c) and we used this to calculate the injection volume via a custom MATLAB analysis. The mean injection volume was 46.5 µL (SD: 10.6 µL) for the MAP gel and 55.2 µL (SD: 6.1 µL) for the hyaluronic acid (Fig. 2b), which approximated the planned 50 µL volume for adequate rabbit vocal fold augmentation. By 3 months, both biomaterials were indistinguishable by MRI from the surrounding tissue (i.e., they had lost the intense T2 signal) (Fig. 2d). We believe this is likely due to tissue infiltration and protein accumulation. Due to its physiological significance, we chose to focus on airway volume as a measure of tissue augmentation and, indirectly, as a measure of material permanence. The airway volume was calculated in the axial dimension from the petiole of the epiglottis to the bottom of the cricoid cartilage for each animal at each time point. Notably, we saw significant differences between MAP and hyaluronic acid starting as early as 3 months and the MAP treatment group maintained approximately the same airway volume for 12 months (1.03-fold change from Day 0), while the airway volume increased at each time point for the hyaluronic acid group indicating loss of tissue augmentation and likely degradation of the material (Fig. 2e) using a custom MATLAB script that calculated the total volume from the cross-sectional area of the 21 MRI slices in this region considering the MRI resolution in the x, y, and z dimensions. Due to the loss of discrete T2 signal intensity observed starting at 3 months, we were unable to directly determine if the MAP implant was remaining in the vocal fold with MRI.

### Quantification of functional vocal fold augmentation

**Fig. 3:**
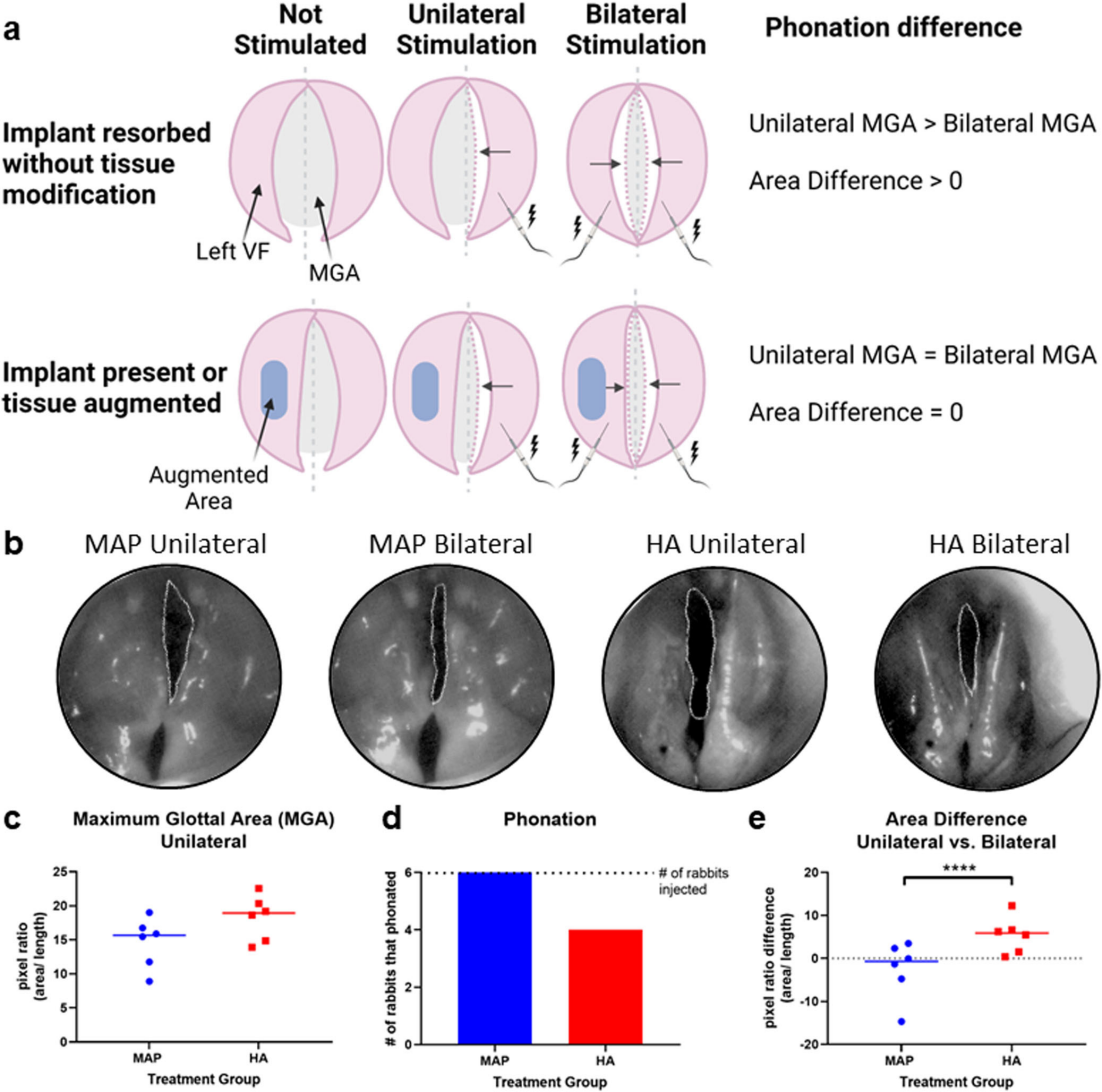
Vocal function measurements. **a** Vocal fold function was assessed using unilateral and bilateral stimulation of the vocal folds. It is anticipated that if an implant is no longer augmenting the tissue, the unilateral stimulation MGA would be greater than the bilateral stimulation MGA. If the implant can compensate for glottic insufficiency, the unilateral and bilateral MGA should be close to equal. **b** Representative bilateral and unilateral phonation images for the two treatment groups with the MGA outlined. **c** MGA measurements for unilateral stimulation for the two treatment groups. **d** In addition to measuring the MGA, we also assessed the ability to phonate as a binary metric and found all MAP gel rabbits produced phonation. **e** As a final functional assessment, the area difference was quantified, and we found the MAP group better compensated for glottic insufficiency as evidenced by a pixel area difference closer to 0. Data is represented by a bar at the median value. ****p < 0.0001. Statistics: Kruskal-Wallis rank sum test. *n* = 6.

The maintenance of augmentation for over a year was promising (Fig. 2), but a clinically relevant vocal fold implant must also be able to restore vocal function. At the 14-month endpoint, we employed a bilateral and unilateral phonatory analysis previously shown to produce glottic insufficiency^17^ in the rabbit model (Fig. 3a). Briefly, a muscle stimulator is used to deliver bipolar square wave pulses to each cricothyroid muscle in order to stimulate glottic closure, with heated and humidified airflow delivered to the glottis to generate an in-vivo evoked vocalization. Endoscopic high-speed video recorded at 5000 frames per second was used to document the amount of glottic insufficiency during consecutive phonation cycles (~400 Hz) with unilateral stimulation (right), mimicking a left vocal paralysis, and bilateral stimulation as an internal control for each rabbit (Fig. 3b). All six (100%) MAP gel treated rabbits were able to achieve phonation in the injury model (unilateral stimulation), while only four of the six (66%) HA rabbits were able to achieve phonation in the injury model (Fig. 3d). The inability to achieve any phonation in our animal model is associated with large glottic gaps. For each rabbit, the length-corrected unilateral maximal glottal area^21–25^ (MGA) (Fig. 3c), representing glottic insufficiency, was subtracted from the bilateral MGA to give an area difference. The area difference was the reproducible output metric, as it allows for each rabbit to serve as their own control, normalizing for biologic variability between rabbits and differences in laryngeal exposure during in vivo phonation. The area difference value reveals the extent of tissue compensation in the setting of glottic insufficiency, where a greater area difference indicates poorer compensation. Perfect compensation for glottic insufficiency would provide an area difference value of 0, indicating successful tissue compensation for effective vocalization. The median area difference for MAP gel-treated rabbits was −0.68 (IQR: −3.88–1.75) while the HA rabbits demonstrated an increase of 5.87 (IQR: 2.51–6.53). A *p*-value of 0.025 between the two groups shows statistical significance (Fig. 3e). On outlier analysis, one MAP rabbit with a value of −14.69 was excluded. After removing this outlier, the median area difference for the MAP gel-treated rabbits was −0.04 (IQR: −1.31–2.35) with a significance of *p* < 0.0001. These results indicate MAP-treated rabbits can better compensate for glottic insufficiency compared to HA rabbits for greater than a year after injection.

### Characterization of tissue *de novo* formation in MAP-treated rabbits

**Fig. 4:**
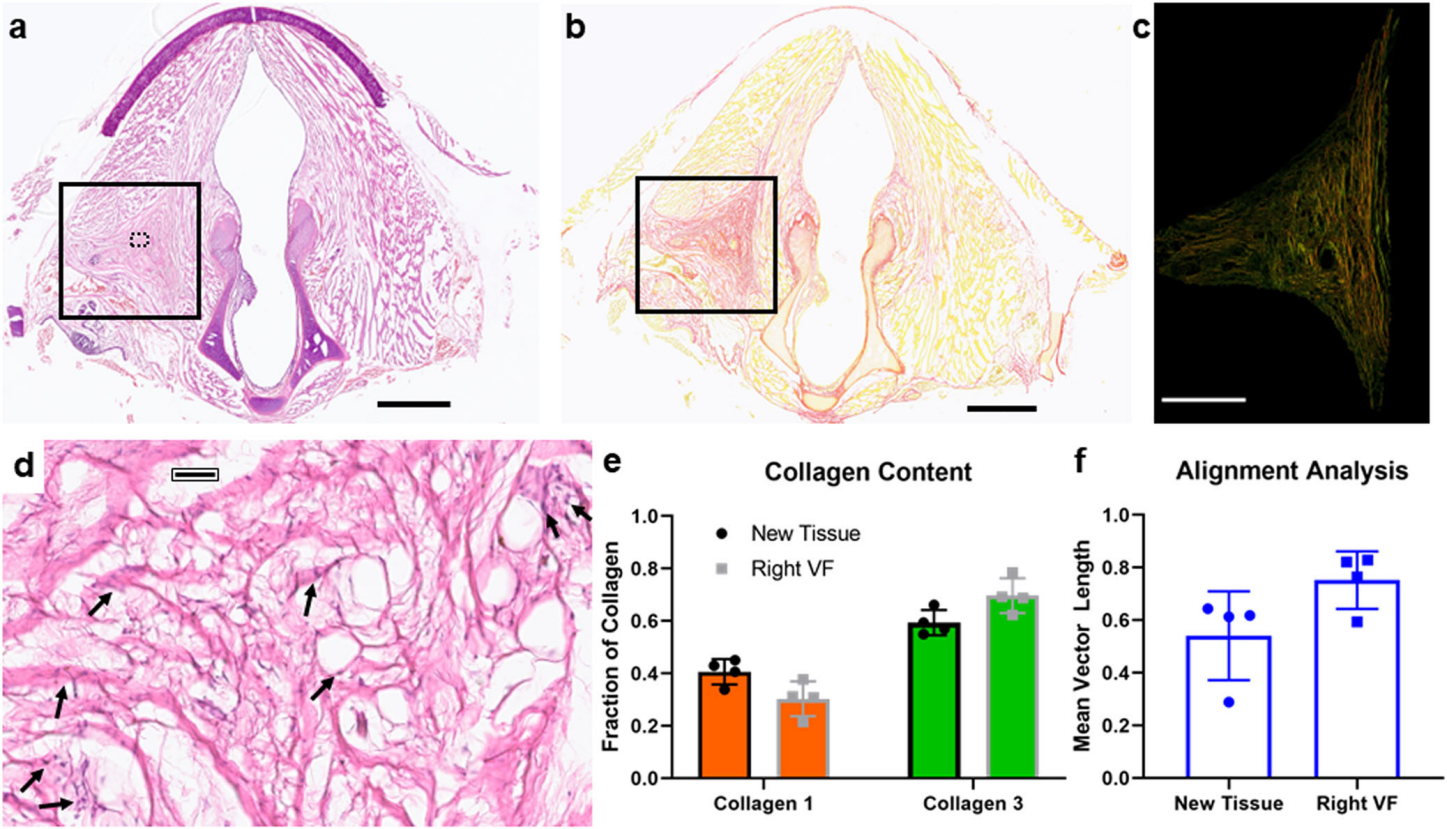
Tissue formation *de novo* in MAP rabbits. **a** H&E image of MAP gel injected rabbit at 14 months. Solid box indicates the area of new tissue formation. Dotted box indicates location of inset displayed in **d** Scale Bar: 2 mm. **b** Brightfield picrosirius red staining of the same MAP gel rabbit. Scale Bar: 2 mm. **c** Polarized light picrosirius red image of the new tissue formed. Scale bar: 1 mm. **d** Zoomed in region of the new tissue. Arrows point to fibroblasts secreting ECM, including wispy mucin and dense, thickened strands of collagen. Scale bar: 50 µm. **e** Collagen type analysis of the new tissue formation compared to the entire right vocal fold. **f** Collagen alignment analysis of the new tissue formation to assess the level of fibrosis. Data is represented as mean+/− standard deviation. Statistics: Paired student’s *t*-test. *n* = 4.

Given the combination of MRI evidence of maintained tissue augmentation (Fig. 2e) and our past published results at 6 months^15^, we anticipated the MAP implant would still be present at 14 months. However, upon H&E staining of the isolated tissue, we found varying levels of degradation (Supplementary Fig. 8). Only two of the rabbits still had significant MAP gel remaining. Surprisingly, three of the rabbits had no discernible MAP gel remaining, and one had just a few microgels visible. The volume previously occupied by the MAP gel had been completely replaced by new tissue *de novo* (Fig. 4a).

In contrast to this novel finding, previous injectable fillers have not maintained augmentation beyond material degradation^13,26,27^. Additionally, biomaterial scaffolds designed to promote tissue bulking have done so by creating intentional collagen-1 deposition and fibrosis^10,11,26^. This led us to further characterize the new tissue formation for collagen content using histopathologic analysis and picrosirius red staining (Fig. 4b, c). In the four rabbits with new tissue, histopathologic analysis confirmed the presence of fibroblasts within the newly formed extracellular matrix (ECM)/ connective tissue. Notably, the ECM components consisted of wispy mucin and dense, thickened collagen bundles directly adjacent to the fibroblasts. Given the vastly different structural differences between this ECM in the wounded vocal cords and the normal connective tissue of unaffected vocal cords, these findings indicate that local fibroblasts present within this ECM are most likely responsible for new extracellular matrix (ECM) deposition in the area of hydrogel injection (Fig. 4d, Supplementary Fig. 11). The new tissue found in place of the MAP gel is highly collagen rich, but has more non-fibrotic (e.g., collagen-3) than fibrotic (e.g., collagen-1) present, and was not significantly different in relative ratios of fibrotic to non-fibrotic composition than the contralateral right vocal fold on the same rabbit (Fig. 4e). Notably, this collagen-rich tissue is different from the surrounding muscle which would be expected in this location due to injection into the thyroarytenoid muscle^28^. To corroborate this observation and confirm it was not fully fibrotic, we performed analysis of collagen fiber alignment, an established hallmark of fibrotic scarring^29–31^, on the new tissue using a published methodology^32^ and saw the mean vector length was 0.54 (Fig. 4f). The mean vector length characterizes fiber alignment and has previously been used to characterize fibrosis^29,32^, where a value of 1 is completely aligned and more fibrotic, while 0 is not aligned. The low level of alignment we observed confirms that this new tissue was primarily non-fibrotic, which makes this finding unique from previous reports of in situ tissue formation^10,11^.

### Characterization of inflammatory response to MAP-treated rabbits

To assess the inflammatory and foreign body response to the implants, a pathologist performed histologic evaluation to determine response to the implants based on FDA guidelines (Supplementary Table 1). Notably, all MAP-treated rabbits had a score of 0 or 1, which denotes 0–2 inflammatory cells per high power field, which is consistent with a minimal acute or chronic inflammatory response. These immune cells were mononuclear with the histological appearance of histiocytes (macrophages). No foreign body response, encapsulation, or calcification of material or adjacent tissue was observed in any specimen, regardless of treatment group (Supplementary Table 2). As a follow-up metric to confirm the immune response in rabbits treated with MAP gel or HA, we stained samples for CD11b, a marker of myeloid cells, including rabbit macrophages/histiocytes. CD11b staining confirmed the presence of rare cells of the myeloid lineage within and adjacent to residual MAP particles, as cells within the CD11b^+^ myeloid cells. Taken together, the histology and immunofluorescent microscopy results confirm MAP gel elicits a negligible/minimal immune response in either (1) vocal cord tissue previously treated with MAP with no MAP remaining 14 months after implantation, and (2) rabbits with residual MAP present 14 months after implantation (Supplementary Figs. 14, 15).

## Discussion

Tissue bulking is desired in many clinical applications where a deficit of patient tissue inhibits normal function, including glottic insufficiency, the specific focus of this study^13,33^. The ideal biomaterial approach would be a resorbable scaffold capable of promoting permanent *de novo* tissue formation that extends beyond the material lifetime.

We developed a unique formulation of MAP hydrogel based on minimally degradable chemistry (i.e., lacking moieties designed to be susceptible to hydrolytic or enzymatic cleavage) to create a long-term MAP implant for glottic insufficiency. Our goal for this study was to create an injectable biomaterial filler that lasted longer than the current clinical standard, hyaluronic acid. We validated the permanence of the scaffold via MRI imaging (Fig. 2) and observed improved functional outcomes in MAP-treated rabbits compared to hyaluronic acid-treated rabbits (Fig. 3). At the 14-month timepoint, in an unprecedented finding, we observed the disappearance of the implant in all but two of the MAP-treated rabbits, and the presence of newly generated non-fibrotic tissue *de novo* at the site of the hydrogel implant (Fig. 4).

We also evaluated whether rabbits implanted with MAP scaffolds for 14 months displayed evidence of an acute or chronic inflammatory response via pathologic scoring and confirmation with CD11b staining. No MAP- or HA-treated rabbits displayed immune cell infiltrates that would be consistent with a sustained inflammatory response via pathologist scoring (Supplementary Table 2). Importantly, evaluating the two rabbits with significant residual MAP present revealed no fibrotic encapsulation, foreign body response, or calcification, phenomena that commonly occur in response to long-term implanted materials 14-months post-implantation (Supplementary Fig. 8). Significant immune cell infiltration was not apparent, with only minimal macrophages within the tissue containing residual implants (similar to previously reported results at 4- and 6-months^15^). CD11b staining confirmed the presence of these rare myeloid cells within and/or surrounding the MAP particles (without forming any considerable immune cell aggregates) in the rabbits with residual MAP (Supplementary Figs. 13, 14). These findings support the idea that immune-mediated proteolysis or destruction is unlikely to be the main process contributing to material resorption. While fully PEG-based formulations are minimally degradable, they can be degraded by reactive oxygen species over time^34^. Reactive oxygen species are present naturally in quiescent tissue^35^ and we hypothesize that this is the likely cause of MAP degradation in this study.

Due to this study consisting of one time-point we cannot determine the timeline of degradation of the material; however, based on our previous study where we observed no appreciable disappearance of the MAP gel at 6 months^15^, and the remaining MAP gel in two of the six animals in this study, we can assume it is longer than 6-months. Further studies are needed to quantify the full degradation timeline and determine the source of variability in MAP resorption observed in this study. Finally, we expect that exploring the mechanism of *de novo* tissue formation will require identification of the participating cell phenotypes and their respective activity (e.g., proteomic analysis of dynamically accumulating proteins).

In conclusion, we have developed a MAP hydrogel formulation that can be delivered via standard clinical apparatus that provides long-term vocal fold augmentation in a rabbit paralysis model for 14 months post-treatment based on MRI at 3, 6, 9, and 12 months and functional measurements at 14 months. The comparison of our optimized MAP gel with the leading clinically utilized injectable, the hyaluronic acid-based Restylane™, demonstrated superior vocal fold reconstruction and excellent functional rehabilitation. Despite seeing varying levels of MAP gel degradation at 14 months, including full resorption for most samples, tissue augmentation, and airway volume was maintained in all MAP gel-treated rabbits with a corresponding benefit to function. Importantly, for the MAP gel-treated rabbits that experienced significant levels of degradation, new tissue had replaced the volume vacated by the material. The ability of a biomaterial to create new tissue that is no more fibrotic than the surrounding tissue has the potential to transform the field of tissue bulking and allow permanent soft tissue augmentation. We believe this material has high translational potential for the treatment of glottic insufficiency because of the ease of clinical use and favorable long-term tissue properties generated.

## Methods

### Animals

All animal research was performed in compliance with the US National Research Council’s Guide for the Care and Use of Laboratory Animals, the US Public Health Service’s Policy on Humane Care and Use of Laboratory Animals, and the Guide for the Care and Use of Laboratory Animals. All animal experiments performed in this study were performed in accordance with the ethical guidelines of the University of Virginia Institutional Animal Care and Use Committee (ACUC) (protocols #4165, #4194).

### Sources of Materials

4-arm 10 kDa PEG-maleimide and 4-arm 10 kDa PEG-thiol were purchased from Nippon Oil Foundry (Japan). RGD cell adhesive peptide (RGDSPGDRCG) was purchased from Watson BioSciences. MethMal annealing macromer was synthesized with 4-arm 20 kDa PEG-maleimide purchased from Nippon Oil Foundry (Japan). Briefly, the PEG-Maleimide was reacted with 2-aminoethanethiol (Acros Organics) at a 0.67:1 thiol to maleimide molar ratio for 2 h at RT in PBS. DMTMM (Oakwood Chemical) at a 12:1 molar ratio to maleimides and methacrylic acid (Sigma) at a 8:1 molar ratio of maleimides was reacted for 50 min in PBS before being added to the reaction. Triethylamine was added to the reaction at the same molar ratio as methacrylic acid and the reaction proceeded overnight at room temperature. Dialysis was performed (3.5 kDa snakeskin tubing) for 3 days in 4 L of 1 M NaCl (changed 2× daily) followed by water (6 × 1 hr washes). The product was frozen, lyophilized, and analyzed with HNMR.^18^. Heparin sodium salt (Millipore Sigma) was thiolated by dissolving heparin sodium salt in ultrapure water and modified with (3-(2-pyridyldithiol) propionyl hydrazide) (PDPH) (Covachem) with a target 3% modification. The pH was adjusted to 6.5 and then DMTMM was added in a 1:1 molar ratio to heparin repeat units (estimate: 619.49 Da) each day of the reaction. The reaction proceeded 3 days at room temperature and then was dialyzed for 3 days in 1 M NaCl (changed 2x daily) and then 0.01 M NaCl (6 × 1 hr washes) before being frozen and lyophilized. After quantification of heparin thiolation using a PDPH deprotection assay, the heparin was deprotected with 25 mM TCEP (Sigma) for 15 min at room temperature. The deprotected solution was dialyzed in 0.01% trifluoroacetic acid and 1 M NaCl (3 h), then 0.1 M NaCl (2 × 1 hr washes), and then 0.01 M NaCl (2 × 1 h washes) before being frozen and lyophilized^19^.

### No heparin formulation

A PEG-based formulation was created to match 14 kPa stiffness. The final concentrations in the gel are PEG-MAL: 16.32 mg/mL, PEG-SH: 10.27 mg/mL, RGD: 1.08 mg/mL, MethMal: 7.52 mg/mL.

### Heparin formulation

A PEG-based formulation was created with 10 mg/mL heparin to match 14 kPa stiffness. The final concentrations in the gel are PEG-MAL: 18.91 mg/mL, PEG-SH: 12.15 mg/mL, RGD: 1.08 mg/mL, MethMal: 7.52 mg/mL, and Heparin: 10 mg/mL. The final heparin concentration in the gel after synthesis was 2.2 mg/mL.

### Macrogel synthesis and characterization

Chemically identical macro-scale gels were formed to analyze mechanical properties and match the vocal fold tissue mechanical modulus, which we previously determined to be 14 kPa^15^. Compressive testing was performed using an Instron at a rate of 0.5 mm/min for 1 min and BlueHill software analyzed the load (N) and extension (mm). Stress-strain curves were generated and Young’s modulus (Pa) was calculated using MATLAB^15,19^.

### Microgel synthesis and characterization

Microgels were created using an overhead spinning method^15^. Briefly, the aqueous pre-gel solution was infused into spinning mineral oil at 2.5 mL/hr with a syringe pump. The oil was supplemented with 1% SPAN-80 and triethylamine (20 µL/mL of gel) and spinning at 350 rpm. Following microgel synthesis, microgels were purified with mineral oil and PBS, sterilized with 70% IPA, and transitioned to sterile PBS in a biosafety cabinet^15^. Microgels were synthesized with Alexa Fluor 488 maleimide to allow for fluorescent identification and imaged using a Molecular Devices ImageXpress. Particle sizing was calculated custom thresholding module (ImageXpress)^15^.

### Microgel pore size quantification

A 10 µL thin annealed MAP scaffold (10% heparin) (*n* = 4) on a slide was imaged with a Zeiss LSM 710 Multiphoton Confocal system located in the Advanced Microscopy Facility at the University of Virginia. The scaffold was infused with 100 µg/mL Texas Red dextran (75 kDa) to visualize the pores. Using the 2-photon setting, a z-stack was imaged that was 150 µm thick with step sizes of 1 µm (for visualization in Fig. 1d) or 10 µm (for quantification) to visualize the particles in green and pores in red. Imaris software was used to create 3D renderings and analyze the average pore area from 2D slices. Prior to analysis, Imaris software was used to clean up the images by performing background subtraction, a gaussian filter, and normalize layers. Briefly, every 10 µm (15 2D slices per scaffold), a surface was created in Imaris software. Using the surface tools, an intensity threshold was applied to outline the pores (Supplementary Fig. 2). Once the threshold was determined, all slices were processed, and the pore area was quantified. All pore measurements were placed in Prism and outlier analysis (ROUT, Q = 1%) was performed prior to data analysis and visualization. Results are reported as a median pore area and standard deviation.

### Microgel preparation for surgery

Heparin and no heparin microgel populations were mixed to yield a 10% heparin scaffold mixture (e.g., 10 µL of heparin microgels combined with 90 µL of no heparin gels). The MAP scaffold mixture was then mixed 1:1 with 40 µm Eosin-Y in PBS and allowed to incubate for at least 10 min. Next, the microgels were centrifuged at 4696 *g* for 5 min to pellet the gel, and the photoinitiator solution was removed. Microgels were then loaded in 1 mL syringes which could be used in surgeries. All preparation was performed in a biosafety cabinet.

### Leporine vocal fold paralysis model

All animal surgeries were performed in accordance with Animal Care and Use Committee guidelines under UVA Animal Protocol 4194. Female New Zealand white breeder rabbits (4–6 kg) were used for all studies. Rabbits were induced with a combination of ketamine (50 mg/kg) and xylazine (5 mg/kg) administered intramuscularly. Spontaneous ventilation with intramuscular sedation was sufficient for injection augmentation procedures and MRI studies. General anesthesia with oral/tracheal intubation using isoflurane (2–2.5%) was used for more invasive procedures including laryngeal nerve resection and phonation testing. Rabbits underwent left recurrent laryngeal nerve resection including a 2 cm segment excision, with cut ends buried into the adjacent muscle to prevent spontaneous regrowth. Each animal received a pre-operative and 6-h post-operative subcutaneous injections of buprenorphine (0.03 mg/kg) for short-term pain relief. For longer-term analgesia, animals received a Fentanyl dermal patch (25 µg) for 3 days. The left hemilaryngeal musculature was allowed to reach maximal atrophy over a 12-week period. After the denervation period, endoscopic injection augmentation (50 µL) of the left vocal fold was performed to restore glottic competence with a 25 cm long 27G laryngeal needle. The rabbits were injected in the level of the true vocal fold with 5 mm depth in the vocalis muscle as measured by depth markings on the injection needle. The treatment groups include saline as a negative control (*n* = 2), hyaluronic acid (Restylane-L, *n* = 6), and vocal MAP gel with heparin microislands (*n* = 6). The MAP gel was annealed using a 300-W xenon light source and fiber optic light cable (KARL STORZ Endoskope) for 60 s with a 2.7 mm 30-degree Hopkins rod rigid endoscope 27018BA (KARL STORZ Endoskope) held 10 mm away from the tissue at 100% power. Laryngoscopic photo documentation was performed using an Image 1 S1 endoscopic camera and AIDA digital capture device (KARL STORZ Endoskope). Rabbits were monitored over 14 months prior to sacrifice. Rabbits were excluded (*n* = 6) from the study if they exhibited airway distress, or unrelated injury or wound complications. All rabbits excluded were not associated with the treatment, except one rabbit that had a local wound infection following injection augmentation with MAP gel. MAP gel is prepared with aseptic technique but is not a sterile preparation. Both saline and hyaluronic acid injections were sterile preparations typically used in human clinical settings.

### Tissue processing

After phonation rabbits were humanely euthanized with pentobarbital intravenous injection. The larynges were isolated and fixed in 10% formalin. After fixing, the larynx was embedded in OCT compound and stored in a −80 °C freezer until sectioning. Prior to sectioning the OCT blocks were equilibrated to −20 °C overnight. Tissues were sectioned into 10 µm and 20 µm thick sections using a cryostat (Cyrostar NX50; ThermoFisher Scientific). Slides were stored at −20 °C until staining.

### Volume Retention Measurements

Rabbits were scanned at day 0, 3 months, 6 months, and 12 months post-treatment using a 3-Tesla Siemens Prisma dedicated research MRI scanner with two orbit surface coils placed in paramedian position on the ventral neck. T2 axial slices were used to track volume measurements over the 12-month period. In the day 0 MRI images, the volume of the injection was measured using a custom MATLAB script (Supplementary Fig. 6) as the injection sites showed very high signal intensity due to high water content. To track the volume a custom MATLAB script was also used to segment the airway area in each axial image from the petiole of the epiglottis to the bottom of the cricoid cartilage for a total of 21 slices to calculate a displacement volume (Supplementary Fig. 6). Total volume was calculated by adding the individual segmented volumes of all 21 slices. A volume was calculated for each slice by multiplying a number of pixels in the cross-sectional area by the x, y, and z resolutions (x-0.3906, y-0.3906, z-0.600 mm/pixel) embedded in the MRI file to get a volume in microliters for each slice. Fold change in airway area for each rabbit from day 0 was calculated.

### Vocal Function Measurements

The phonation capability of the rabbits was analyzed at the 14-month endpoint to determine functional benefit. After a plane of general anesthesia was achieved, dissection was carried down to expose the trachea and a distal tracheotomy was performed and a size 3.0 endotracheal tube was placed for ventilation and isoflurane gas anesthesia. The rabbit was placed in laryngeal suspension using a ring stand and a rabbit-modified pediatric Parsons laryngoscope. A second, more proximal tracheostomy was placed two tracheal rings above the ventilation tube, and a size 3.5 endotracheal tube was placed in retrograde fashion to deliver heated (37 °C) and humidified (100%) oxygen (at 10 L/min) to the glottis. The cricothyroid muscles were isolated bilaterally, and pairs of 0.008” coated stainless steel anode-cathode wire electrodes (A-M Systems, part #791400, Sequim, WA) were inserted into each cricothyroid muscle. A pulse stimulator (A-M Systems, Model 2100, Sequim, WA) was used to deliver square wave pulses to each cricothyroid muscle. Vocal fold approximation for phonation was stimulated using a 60-Hz, 80-mA bipolar stimulus bilaterally in series configuration with train of 1-ms pulses, with the stimulus of 3 s on followed by 3 s off. Unilateral stimulation producing glottic insufficiency was performed with a 40-mA stimulation to only the right cricothyroid muscle. Several laryngeal vibratory cycles were captured over 10 s using a high-speed camera (Fastec IL-5; Fastec Imaging, San Diego, CA) and an endoscopic zoom coupler (KARL STORZ Endoskope) in combination with a 30° rigid telescope (KARL STORZ Endoskope). Video parameters included recording at 5,000 frames per second in monochrome color, shutter speed of 187 microseconds, and a gain of 1.0 with a spatial resolution of 256 × 512 pixels. 2X binning and 10-bit image settings using FasMotion software version 2.5.3 (Fastec Imaging, San Diego, CA). Video analysis was performed in a blinded fashion using ImageJ software (National Institutes of Health, Bethesda, MD). Three independent raters experienced in laryngoscopy interpretation (HLK, WMK, JJD) measured maximal glottal area (MGA) across five consecutive glottic cycles during sustained periodic phonation for each rabbit in bilateral and unilateral protocols. Pre-rating consensus training was performed among the raters to standardize the measurement protocol. Five of 24 measurements demonstrated a coefficient of variation greater than 15. These outlier images were reviewed in a consensus conference, and differences in interpretation were discussed. The images were then re-measured independently and resubmitted for analysis. The primary vocal function endpoint difference in MGA was calculated by determining the difference in area between unilateral stimulation (injury model) and bilateral stimulation serving as an internal control (paralysis bypassed with direct muscle stimulation), with each measurement corrected for the vocal fold length (Supplementary Fig. 5).

### Histology

Hematoxylin and Eosin (H&E) staining was performed using a previously established protocol for frozen tissues^36^. Picrosirius Red staining was performed following manufacturer’s protocol (Abcam). Due to tissue fragility, the thyroid cartilage was removed on sections to produce cleaner stains (i.e., less folds) (Supplementary Fig. 7). CD11b staining was performed using the same protocol and antibody (Mouse Anti-Rabbit CD11b [Bio-Rad MCA802GA], 1:100 dilution) described previously^15^ Briefly, sections were rehydrated with PBS and incubated with blocking buffer (PBS, 5% BSA, 5% milk, 5% FBS) for 30 min at room temperature. Next, the primary antibody was applied overnight at 4 °C and the slides were washed with PBS before applying the secondary antibody (AlexaFluor 488 Goat Anti-mouse) at a 1:1000 dilution for 1 h at room temperature. Finally, the slides were washed with PBS stained with DAPI, and mounted with Prolong Gold Antifade mountant. H&E and Picrosirius Red brightfield imaging was performed by the University of Virginia Biorepository and Tissue Research Facility (BTRF) core using a Hamatsu Slide scanner. Picrosirius Red polarized light imaging was performed using a LEICA Thunder microscope at the UVA Advanced Microscopy Facility. Polarized light image analysis was conducted using ImgeJ by separating the red and green channels, auto-thresholding each channel, and then measuring the percent area (Supplementary Fig. 12). For this analysis, the new tissue was cropped in each image to analyze the full area and the full right vocal fold was analyzed. Three 10X sections were analyzed for each of the rabbits that had new tissue formation (*n* = 4). Fiber alignment analysis was conducted using a previously published MATLAB method available on github^29,32^ with a square size of 100 and threshold of 10000 (Supplementary Fig. 13). Three 20X images from each vocal fold with new tissue were analyzed using the right vocal fold as a comparison. Three different sections were used for each rabbit (*n* = 4).

### Pathologist Scoring

A standard scoring system was used to assess response to materials based on FDA guidelines (Supplementary Table 1) but modified for a long-term implant by assessing all immune cells together. H&E images were used for scoring. To assess inflammation, the number of inflammatory cells was assessed for 10 high-power fields (400X).

### Statistics

An unpaired student’s t-test was used to compare MAP to hyaluronic acid at each time point for MRI analysis (*n* = 6). A paired student’s t-test was used to compare collagen-1 and collagen-3 content between the new tissue composition and right vocal fold (*n* = 4). Materials characterization, MRI data, and histology statistical analysis was conducted using GraphPad Prism. Statistical analyses of the change in MGA across treatment groups was performed using RStudio version 1.4.1103 (packages “tidyverse”, “dplyr”, “rstatix”). The change in MGA was imported to RStudio, and Kruskal-Wallis rank-sum test was performed (*n* = 6). An eta-squared statistic was calculated to determine effect size (0.337). Outlier analysis was performed, excluding any values more than 1.5 IQRs below the 1st quartile or above the 3^rd^ quartile (1 outlier identified). Individual maximal glottic area measurements were imported, and standard errors of measurement (SEM), and inter-rater and intra-rater reliability statistics were determined through calculation of single score intraclass correlation coefficients (ICC) of consistency, using a two-way random-effects model (package “irr”). The reliability measures were all found to be within excellent agreement range consistent with a precise test. The inter-rater ICC was 0.87 (95% CI 0.84–0.90, F = 21, *p* = 2.34e−108, SEM = 1.71). Intra-rater ICCs were 0.92 (95% CI 0.81–0.97, F = 24.5, *p* = 1.53e−9, SEM = 1.02), 0.94 (95% CI 0.86–0.98, F = 34.4, *p* = 7.27e−11, SEM = 0.94), and 0.75 (95% CI 0.48–0.89, F = 7.09, *p* = 4.12e−05, SEM = 2.34).

### Reporting summary

Further information on research design is available in the Nature Research Reporting Summary linked to this article.

## Supplementary information

Supplementary Information

Reporting Summary

Supplemental Movie S1

Supplemental Movie S2

## Data Availability

The data that support the findings of this study are available from the corresponding authors upon reasonable request.
