# Supplementary material for: De novo tissue formation using custom microporous annealed particle hydrogel provides long-term vocal fold augmentation
Source: NPJ Regen Med. 2023 Feb 23;8:10. doi: 10.1038/s41536-023-00281-8 (PMC9950481; doi:10.1038/s41536-023-00281-8)
Supplement: Supplementary file 1 — Supplementary Information [file 41536_2023_281_MOESM1_ESM.pdf]

Supplementary Information:

De Novo Tissue Formation using Custom Microporous Annealed Particle Hydrogel Provides Long-Term Vocal Fold Augmentation

*Lauren J. Pruett, Hannah L. Kenny, William M. Swift, Katarina J. Catallo, Zoe R. Apsel, Lisa S. Salopek, Philip O. Scumpia, Patrick S. Cottler, James J. Daniero, Donald R. Griffin**

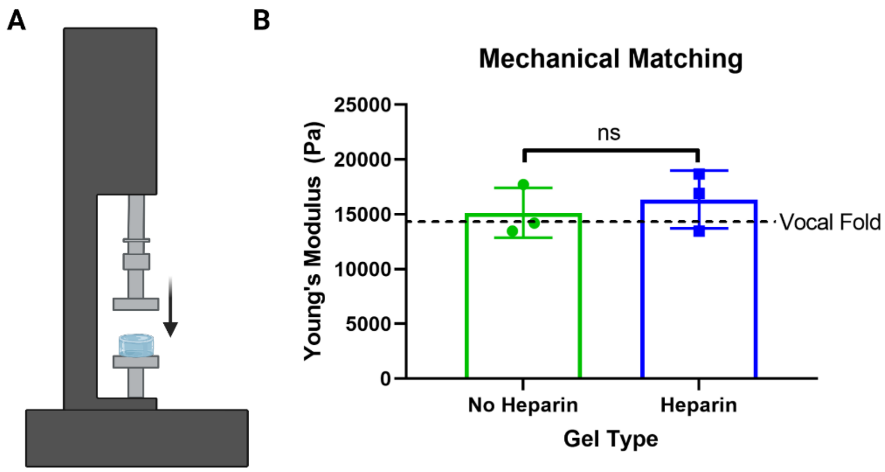

Supplementary Figure 1: Mechanical matching of heparin and no heparin gel. A) Instron testing of macrogels to determine the Young's modulus. B) Heparin and no heparin gels were matched to approximately 14kPa (n=3). Data represented as mean ± standard deviation.

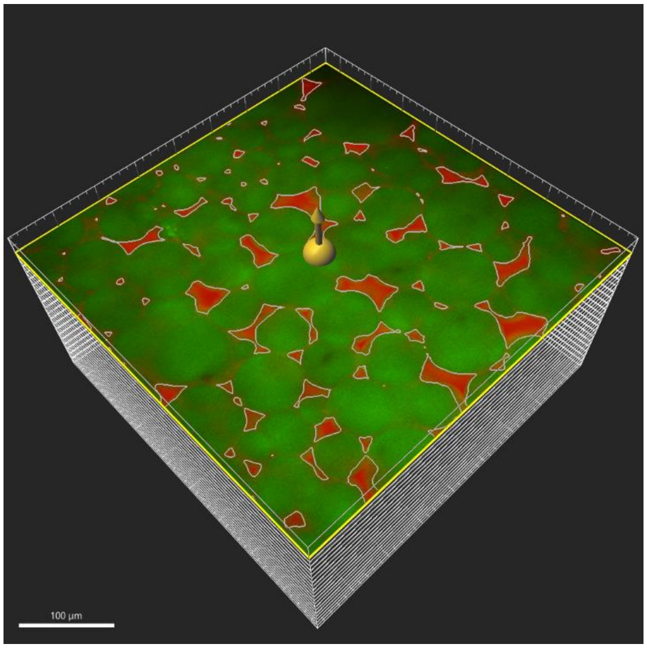

Supplementary Figure 2: Pore area quantification in Imaris software. Using Imaris software, an intensity threshold is applied to each 2D slice to threshold the pores (red) from the microgels (green).

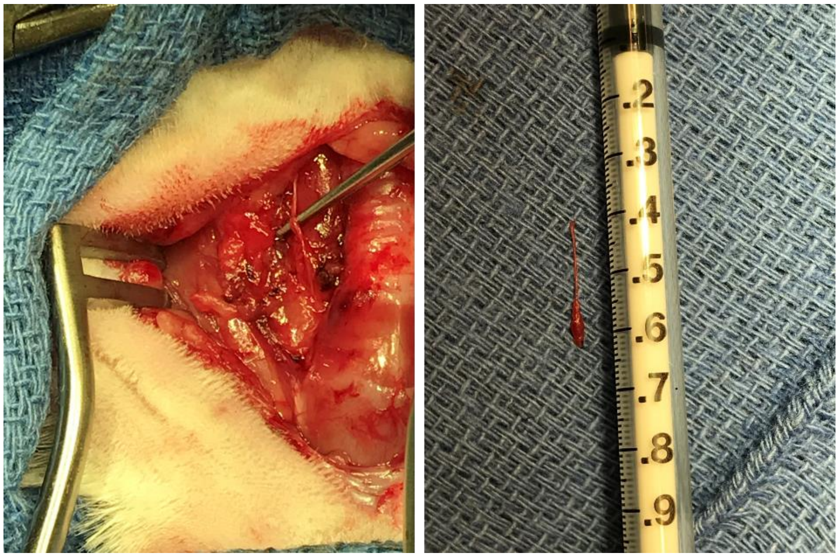

Supplementary Figure 3: Removal of the left recurrent laryngeal nerve to induce vocal fold paralysis.

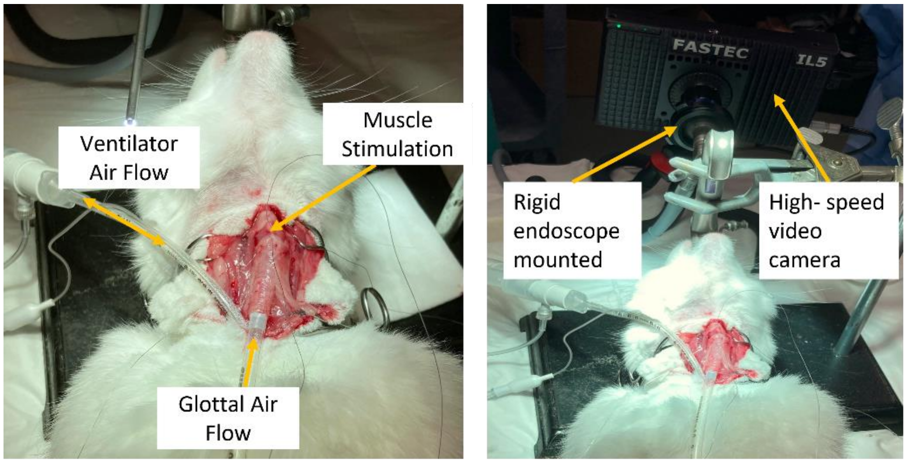

Supplementary Figure 4: Phonation and high-speed recording at the 14-month time point.

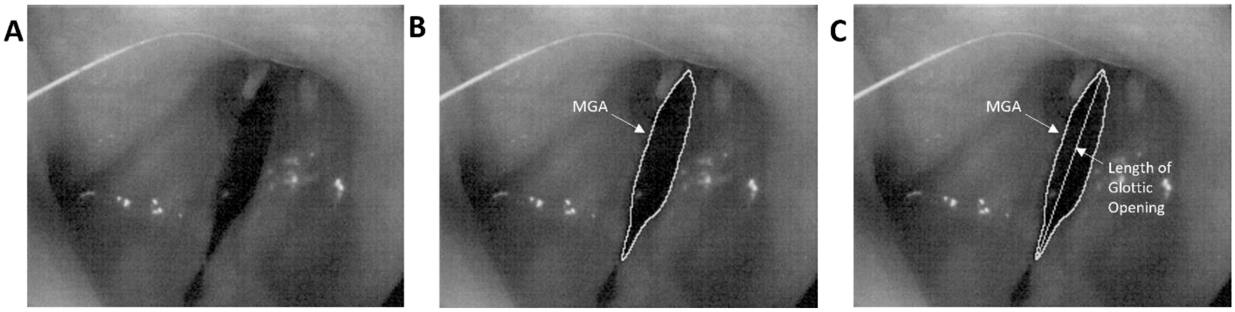

Supplementary Figure 5: Vocal function measurements during stimulated glottic closure. Beginning with A) the endoscopic image of the glottic opening in ImageJ, B) the "freehand" tool is used to outline the area of the glottic opening from the anterior commissure to the vocal process, and C) the "straight" tool is used to measure the glottic length from the anterior commissure anteriorly to the midpoint between the vocal processes posteriorly.

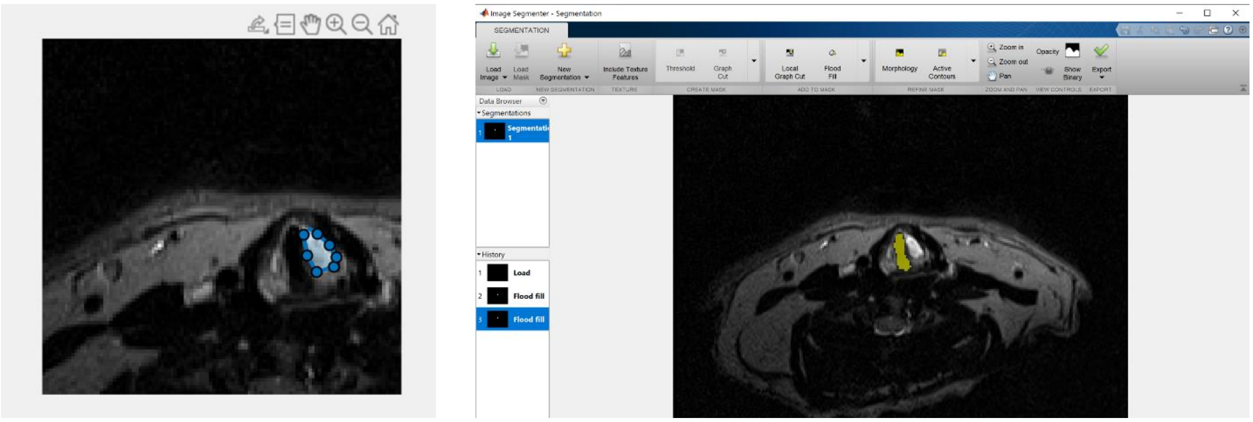

Supplementary Figure 6: MRI Analysis. Injection volume analysis was measured by tracing the bright injection area in MATLAB for each slice it was present. Tracing in MATLAB was performed using the “drawpolygon” function and then measuring the number of pixels in the traced polygon. Airway volume analysis was performed by automatic segmentation using Image Segmenter in MATLAB if possible, and if not, by tracing in MATLAB for a series of 21 consecutive slices from the petiole of the epiglottis to the bottom of the cricoid cartilage. Total volume was calculated by adding the volume of each individual slice considering the resolution in the x, y, and z dimensions (x-0.3906, y-0.3906, z-0.600 mm/pixel).

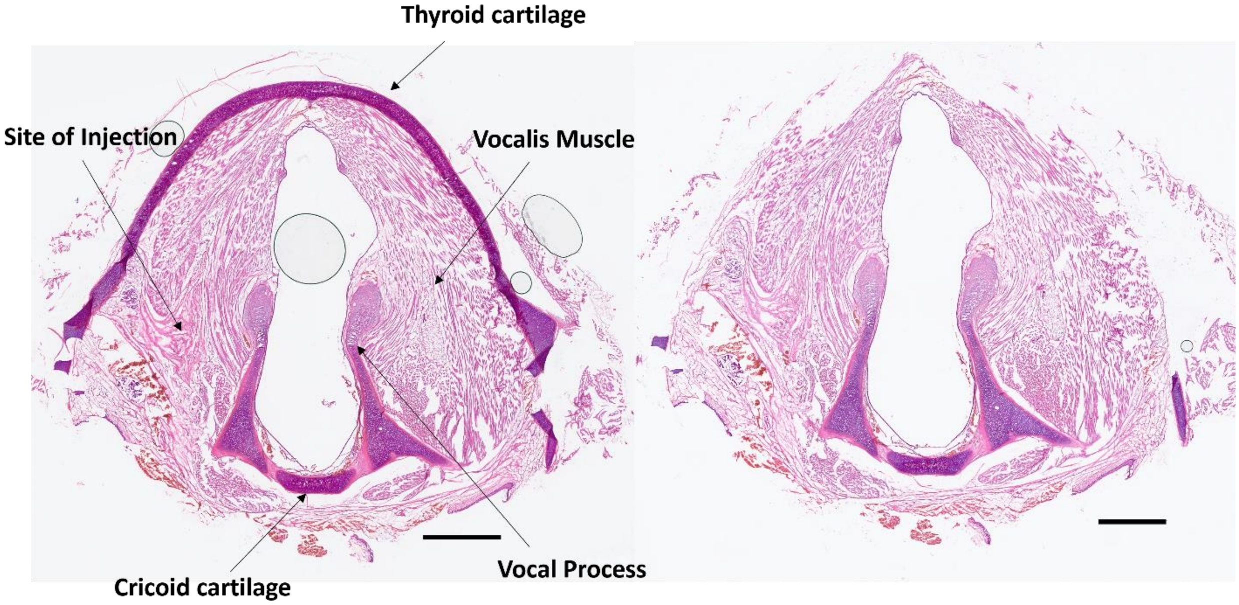

Supplementary Figure 7: Labeled H&E section. A fully labeled H&E section on the left with the thyroid cartilage still in place. To make staining easier (i.e. to prevent folds), the thyroid cartilage was removed as seen in the right image.

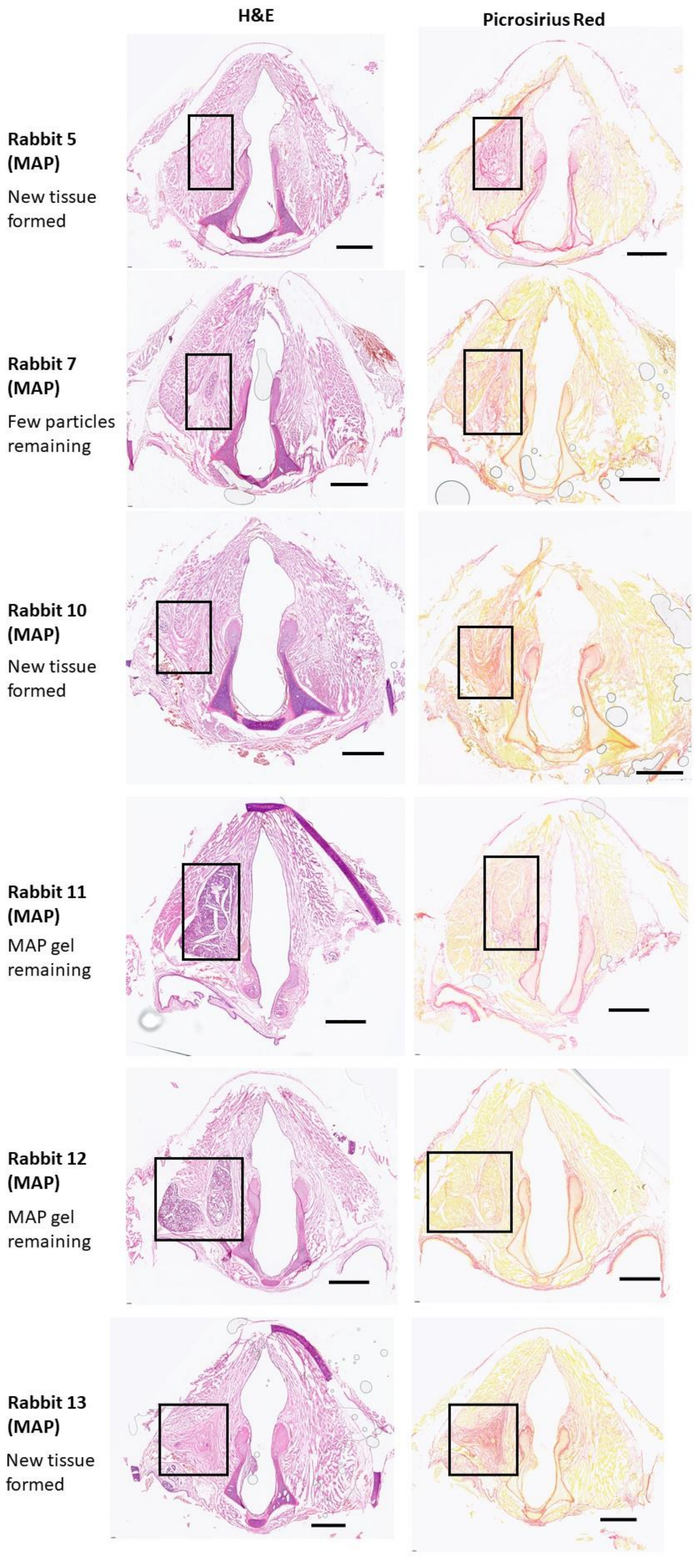

Supplementary Figure 8: H&E and Picrosirius Red images of the MAP gel injected rabbits. Injection was performed on the left vocal fold. MAP injection site is outlined in black box. Scale bar is 2mm.

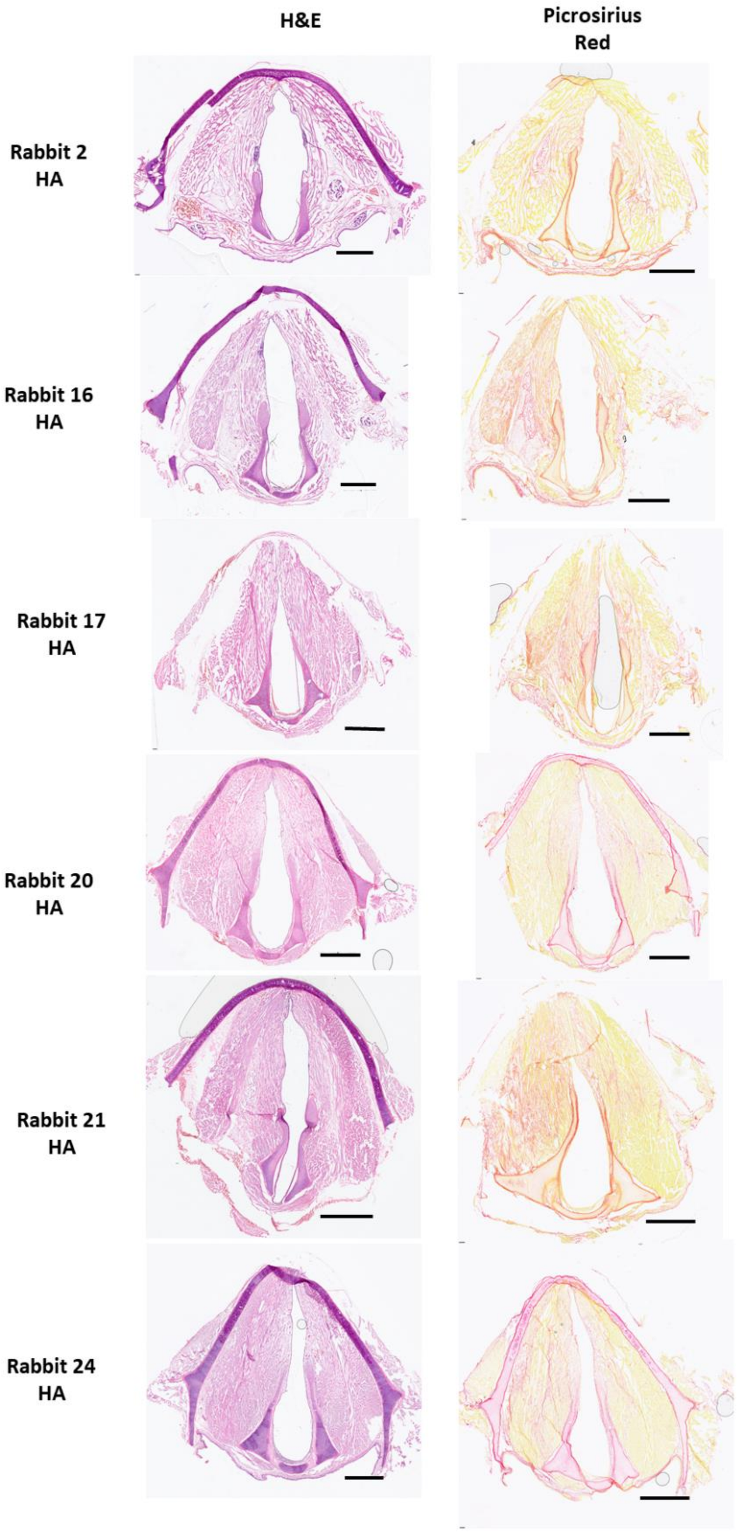

Supplementary Figure 9: H&E and Picrosirius Red images of the HA injected rabbits. Injection was performed on the left vocal fold. Scale bar is 2mm.

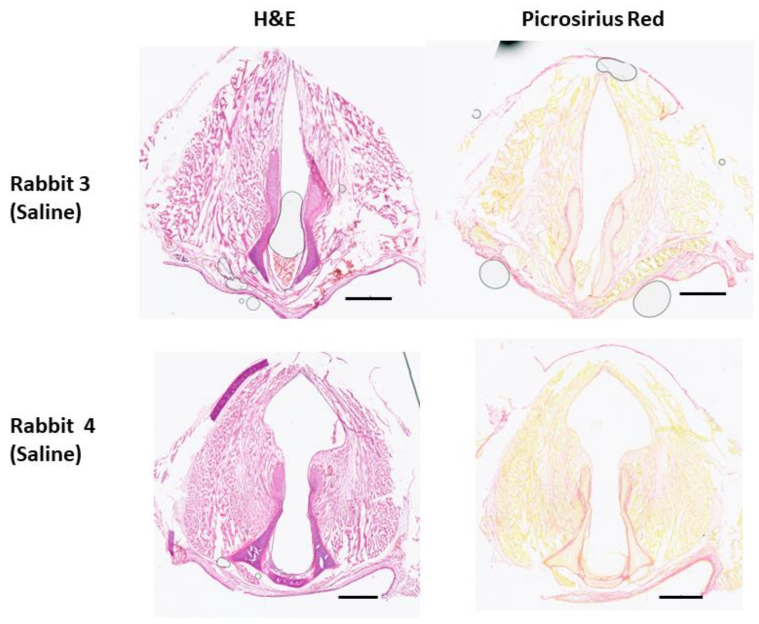

Supplementary Figure 10: H&E and Picrosirius Red images of the saline injected rabbits. Injection was performed on the left vocal fold. Scale bar is 2mm.

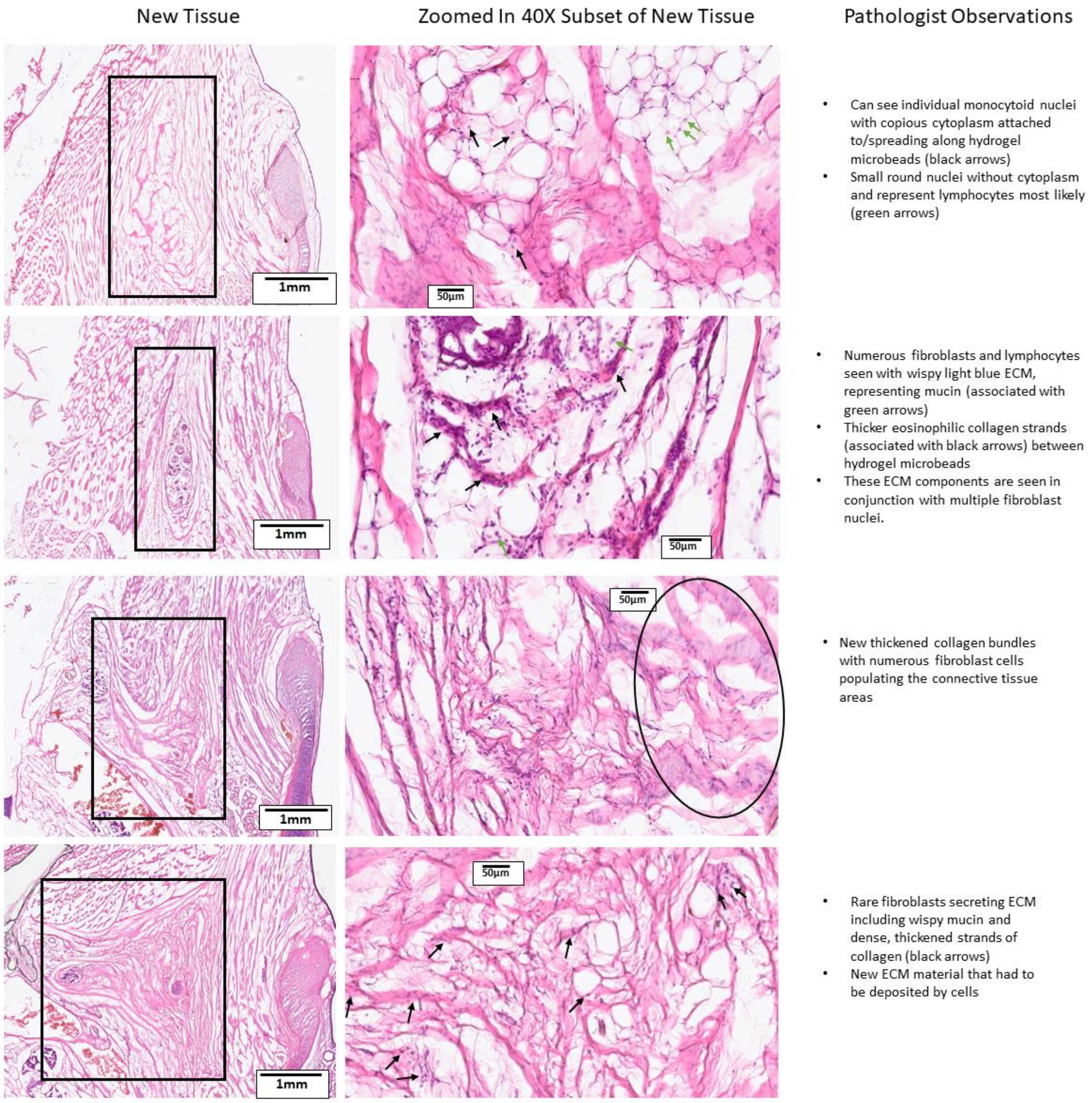

Supplementary Figure 11: 40X H&E representative insets of the new tissue area (right images) were examined by a pathologist to identify cell types populating the area with new tissue (left images). Notably, all tissues had cells present throughout, primarily fibroblasts, and areas of new ECM materials including wispy mucin and collagen strands.

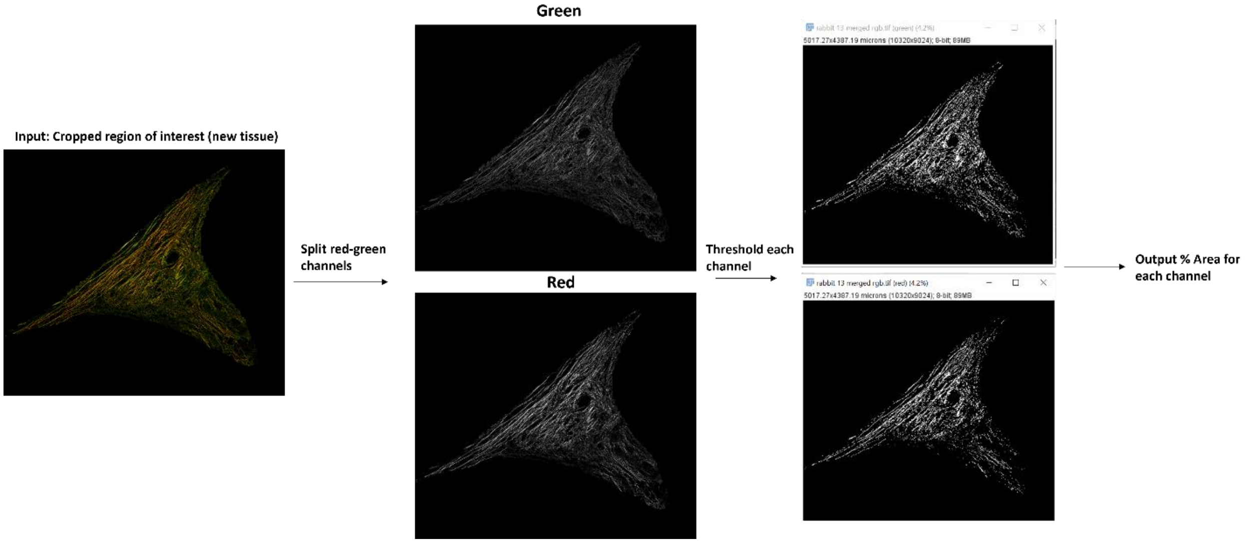

Supplementary Figure 12: Picrosirius red percent area analysis in ImageJ to determine the collagen type composition. Briefly, the red and green image channels are separated, then auto-thresholded to obtain a percent area.

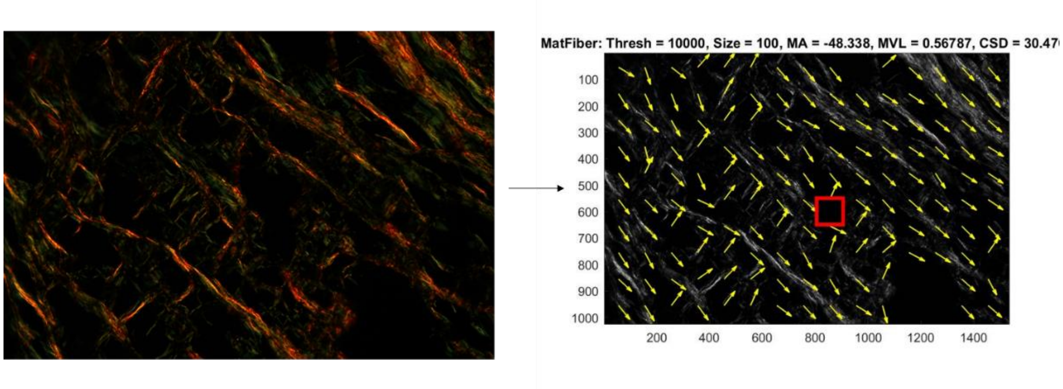

Supplementary Figure 13: Picrosirius red alignment analysis. A 20X polarized picrosirius red image is imported into MatFiber code[32] and outputs MVL as an alignment metric. For all images, a square size of 100 and threshold of 10000 was used. Yellow arrows depict fiber directions. Red square shows size of approximately 1 fiber.

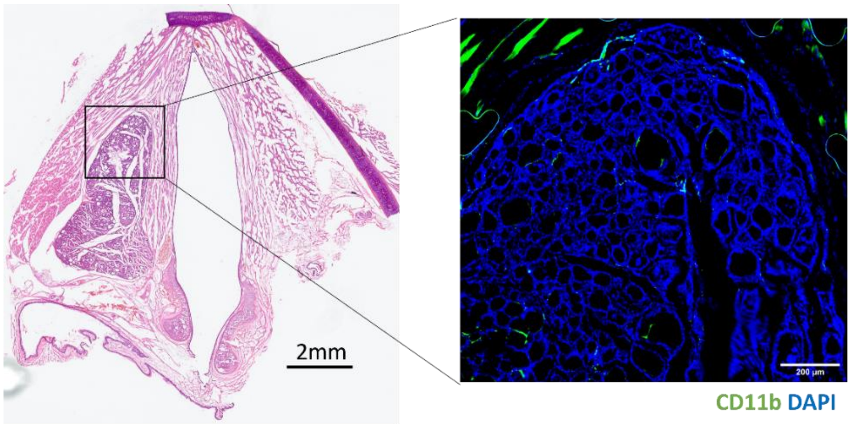

Supplementary Figure 14: CD11b staining of Rabbit 11 which still had a significant amount of MAP gel remaining.

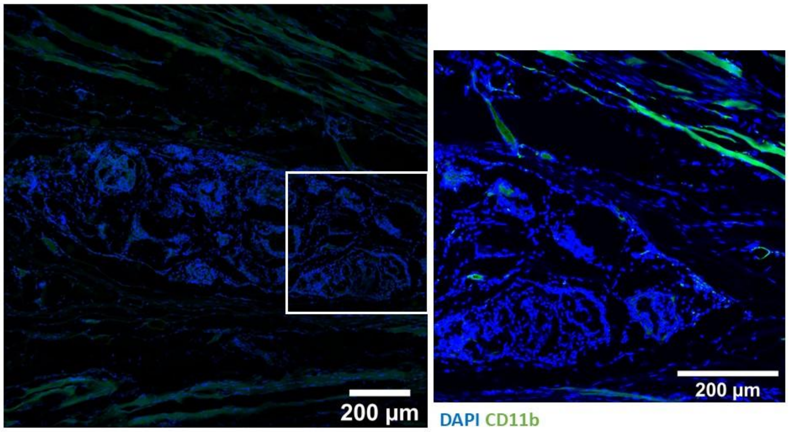

Supplementary Figure 15: CD11b staining of Rabbit 7, which just has several particles remaining.

Supplementary Table 1: Scoring assessment for inflammation and foreign body scoring.

Assessment	Score	Criteria
Acute and Chronic Inflammation Scoring	0	No appreciable immune cells
	1	Average 1-2 immune cells per high power field
	2	2-10 immune cells per high power field
	3	>10 immune cells per high power field
	4	Sheets of immune cells, microabscess, or abscess formation
	5	Sheets of foamy histiocytes with surrounding lymphocytes engulfing material (atypical granuloma formation)
Foreign Body Scoring	0	No appreciable foreign body response
	1	Fibrous encapsulation of material, without histiocytic/macrophage response
	2	Fibrous encapsulation of material with histiocytic/macrophage response, no giant cells
	3	Fibrous encapsulation of material with histiocytic/macrophage response, with some giant cells
	4	Foreign body granuloma formation with brisk inflammatory response, numerous foreign body giant cells with extrusion of material from tissue

Supplementary Table 2: Pathological scoring for each treatment group to assess inflammation and foreign body response via H&E staining.

Rabbit #	Treatment Group	Acute and Chronic Inflammation Score	Foreign Body Score
5	MAP	0	0
7		1	0
10		0	0
11		1	0
12		0	0
13		0	0
2	HA	0	0
16		0	0
17		0	0
20		0	0
21		0	0
24		0	0
3	Saline	0	0
4		0	0
