# Supplementary material for: De novo tissue formation using custom microporous annealed particle hydrogel provides long-term vocal fold augmentation
Source: NPJ Regen Med. 2023 Feb 23;8:10. doi: 10.1038/s41536-023-00281-8 (PMC9950481; doi:10.1038/s41536-023-00281-8)
Supplement: Supplementary file 2 — Reporting Summary [file 41536_2023_281_MOESM2_ESM.pdf]

nature portfolio

Corresponding author(s): James Daniero, MD and Donald Griffin, PhD

Last updated by author(s): Jun 30, 2022

Reporting Summary

Nature Portfolio wishes to improve the reproducibility of the work that we publish. This form provides structure for consistency and transparency in reporting. For further information on Nature Portfolio policies, see our Editorial Policies and the Editorial Policy Checklist.

Statistics

For all statistical analyses, confirm that the following items are present in the figure legend, table legend, main text, or Methods section.

n/a	Confirmed	
☐	☒	The exact sample size (n) for each experimental group/condition, given as a discrete number and unit of measurement
☐	☒	A statement on whether measurements were taken from distinct samples or whether the same sample was measured repeatedly
☐	☒	The statistical test(s) used AND whether they are one- or two-sided<br>*Only common tests should be described solely by name; describe more complex techniques in the Methods section.*
☒	☐	A description of all covariates tested
☐	☒	A description of any assumptions or corrections, such as tests of normality and adjustment for multiple comparisons
☐	☒	A full description of the statistical parameters including central tendency (e.g. means) or other basic estimates (e.g. regression coefficient) AND variation (e.g. standard deviation) or associated estimates of uncertainty (e.g. confidence intervals)
☐	☒	For null hypothesis testing, the test statistic (e.g. F, t, r) with confidence intervals, effect sizes, degrees of freedom and P value noted<br>*Give P values as exact values whenever suitable.*
☒	☐	For Bayesian analysis, information on the choice of priors and Markov chain Monte Carlo settings
☒	☐	For hierarchical and complex designs, identification of the appropriate level for tests and full reporting of outcomes
☐	☒	Estimates of effect sizes (e.g. Cohen's d, Pearson's r), indicating how they were calculated

Our web collection on statistics for biologists contains articles on many of the points above.

Software and code

Policy information about availability of computer code

Data collection	MATLAB R2017b was used to quantify injection volume and airway volume for Figure 2 as described in Figure S6. Video analysis of MGA was performed using ImageJ. Microgel pore size quantification was calculated using Imaris software. Microgel size was analyzed using a Molecular Devices ImageXpress module.
Data analysis	GraphPad Prism 9.1 was used for statistical analysis (t-test) of MRI airway volume, collagen content, and materials characterization. RStudio version 1.4.1103 (packages "tidyverse", "dplyr", "rstatix") was used for stastical analyses of change in MGA.

For manuscripts utilizing custom algorithms or software that are central to the research but not yet described in published literature, software must be made available to editors and reviewers. We strongly encourage code deposition in a community repository (e.g. GitHub). See the Nature Portfolio guidelines for submitting code & software for further information.

Data

Policy information about availability of data

All manuscripts must include a data availability statement. This statement should provide the following information, where applicable:

- Accession codes, unique identifiers, or web links for publicly available datasets
- A description of any restrictions on data availability
- For clinical datasets or third party data, please ensure that the statement adheres to our policy

The data that support the findings of this study are available from the corresponding authors upon reasonable request.

Human research participants

Policy information about studies involving human research participants and Sex and Gender in Research.

Reporting on sex and gender	Human research participants were not used in this study.
Population characteristics	Human research participants were not used in this study.
Recruitment	Human research participants were not used in this study.
Ethics oversight	Human research participants were not used in this study.

Note that full information on the approval of the study protocol must also be provided in the manuscript.

Field-specific reporting

Please select the one below that is the best fit for your research. If you are not sure, read the appropriate sections before making your selection.

☒ Life sciences ☐ Behavioural & social sciences ☐ Ecological, evolutionary & environmental sciences

For a reference copy of the document with all sections, see nature.com/documents/nr-reporting-summary-flat.pdf

Life sciences study design

All studies must disclose on these points even when the disclosure is negative.

Sample size	6 rabbits per group (MAP and Hyaluronic Acid) were used. A sample size of 6 was selected via a power analysis assuming 50% variability in vocal fold injection and an expected resorption rate of 80% greater in the hyaluronic acid group compared to MAP gel.
Data exclusions	Outlier analysis was performed for the MGA measurements, excluding any values more than 1.5 IQRs below the 1st quartile or above the 3rd quartile.
Replication	Due to the length of the study, this study was unable to be repeated.
Randomization	A random number generator was used to select which rabbits received each treatment group.
Blinding	The raters for functional analysis were blinded. MRI analysis was blinded to the treatment group. The pathologist findings were unable to be blinded due to the differences in histological appearance between each group.

Reporting for specific materials, systems and methods

We require information from authors about some types of materials, experimental systems and methods used in many studies. Here, indicate whether each material, system or method listed is relevant to your study. If you are not sure if a list item applies to your research, read the appropriate section before selecting a response.

Materials & experimental systems

n/a	Involved in the study	
☐	☒	Antibodies
☒	☐	Eukaryotic cell lines
☒	☐	Palaeontology and archaeology
☐	☒	Animals and other organisms
☒	☐	Clinical data
☒	☐	Dual use research of concern

Methods

n/a	Involved in the study	
☒	☐	ChIP-seq
☒	☐	Flow cytometry
☒	☐	MRI-based neuroimaging

Antibodies

Antibodies used	Mouse anti-rabbit CD11b (Bio-Rad MCA802GA) - 1:100 dilution
Validation	This antibody has been previously used in 10+ publications in rabbit species as indicated on the product's webpage. We have previously validated this antibody in our previous publication (https://doi.org/10.1002/lary.28442).

Animals and other research organisms

Policy information about studies involving animals; ARRIVE guidelines recommended for reporting animal research, and Sex and Gender in Research

Laboratory animals	New Zealand white breeder rabbits (4-6kg) were used for all studies.
Wild animals	Study did not involve wild animals.
Reporting on sex	Female New Zealand white breeder rabbits were used for all studies.
Field-collected samples	Study did not involve samples collected from the field.
Ethics oversight	All animal surgeries were performed in accordance with Animal Care and Use Committee guidelines under University of Virginia Animal Protocol 4194.

Note that full information on the approval of the study protocol must also be provided in the manuscript.
